# Screening for second primary tumors in the aerodigestive tract in non-Asian populations with head and neck cancer – systematic review and meta-analysis

**DOI:** 10.1016/j.esmogo.2025.100167

**Published:** 2025-04-03

**Authors:** A.D.I. Maan, S.E.M. van de Ven, S. Keereweer, R. Cornelissen, P.D. Siersema, A.D. Koch

**Affiliations:** 1 Department of Gastroenterology and Hepatology, Erasmus MC Cancer Institute, University Medical Center, Rotterdam, The Netherlands; 2 Department of Otolaryngology-Head and Neck Surgery, Erasmus MC Cancer Institute, University Medical Center, Rotterdam, The Netherlands; 3 Department of Pulmonology, Erasmus MC Cancer Institute, University Medical Center, Rotterdam, The Netherlands

**Keywords:** second primary tumor, screening, esophageal cancer, head and neck cancer, lung cancer

## Abstract

**Background:**

Patients diagnosed with a primary tumor in the esophagus, lungs, or head and neck are at an increased risk for developing second primary tumors (SPTs) in these regions. Most studies on SPT prevalence focus on Asian populations, with limited data available for non-Asian groups, leaving the utility of screening unclear. This review aims to assess the yield of screening for SPTs in non-Asian populations following a primary tumor in these regions.

**Patients and methods:**

A systematic literature search was conducted to identify studies on screening for esophageal, lung, or head and neck SPTs after a primary tumor diagnosis in any of these sites. The primary outcome was the prevalence by screening of all diagnosed SPTs in the esophagus, head and neck or lungs.

**Results:**

Due to limited data on screening for SPTs after esophageal or lung tumors, this review focused solely on screening after primary head and neck tumors. A total of 26 studies with 8071 patients from non-Asian countries were included. The pooled prevalence for all SPTs was 5.4% [95% confidence interval (CI) 4.1% to 7.2%]. The pooled prevalence for esophageal SPTs individually was 5.3% (95% CI 3.7% to 7.7%), for head and neck SPTs 4.6% (95% CI 1.0% to 18.1%) and for lung SPTs 4.0% (95% CI 2.6% to 6.2%). Most SPTs were detected in combination with an index hypopharynx carcinoma (60.0%). The proportion of synchronous (45.3%) and metachronous (54.7%) SPTs was similar.

**Conclusion:**

Endoscopic screening for esophageal SPTs in non-Asian countries should be considered, especially in patients with a primary hypopharynx carcinoma.

## Highlights

• Research on the yield of screening for SPTs in the esophagus, head and neck or lungs is scarce in non-Asian populations.
• The pooled prevalence for SPTs in head and neck, esophagus or lungs after a primary HNC diagnosis was 5.4%.
• The proportion of synchronous (45.3%) and metachronous (54.7%) SPTs was similar after a primary HNC diagnosis.
• Screening for esophageal SPTs in non-Asian countries should be considered, especially in primary hypopharynx carcinoma.

## Introduction

The number of cancer deaths has declined over the last years due to earlier detection through screening programs, improvement in diagnostic tests and more effective treatments.^1^^,^^2^ As the survival rate after cancer diagnosis increases, the risk of developing a second primary tumor (SPT) increases simultaneously.^3^^,^^4^ For example, a recently published large Danish study showed an increasing cumulative incidence of SPTs of 6.3% at 5 years and 13.5% at 15 years after initial primary tumor diagnosis.^5^

Tumors that arise in the esophagus, lungs and head and neck are associated with an increased risk of developing SPTs.^4^^,^^6^, ^7^, ^8^ The frequent occurrence of SPTs in these regions is often attributed to the ‘field cancerization theory’, which proposes that normal tissue near the primary tumor bears molecular alterations caused by prolonged exposure to carcinogens such as alcohol and tobacco. These changes predispose the tissue to local recurrences or SPTs over time.^9^^,^^10^ Early detection and the availability of less invasive treatment options for SPTs have the potential to enhance survival rates and improve quality of life for patients with primary tumors in these areas.^11^^,^^12^

Most studies investigating the prevalence of SPTs in the esophagus, lungs and head and neck region have been conducted in Asian populations, where screening for SPTs after curative treatment of the first primary tumor is acknowledged and incorporated into clinical guidelines.^13^^,^^14^ SPT prevalence data outside Asia are scarce and it is therefore unknown whether screening for SPTs is useful in a non-Asian population. Limited available research indicates that the SPT prevalence in non-Asian populations can be as high as 17% in patients with a primary tumor located in the head and neck region, lungs or esophagus.^5^^,^^15^^,^^16^ The purpose of this systematic review is to investigate the yield of screening for SPTs in the esophagus, lungs and head and neck region in non-Asian populations after diagnosis of a primary tumor in any of these sites.

## Patients and methods

### Literature search and selection criteria

In November 2024, a comprehensive literature search was conducted in collaboration with the medical library at Erasmus University Rotterdam, the Netherlands, with no restriction on publication date. The search was conducted in multiple databases, including Medline, Embase and Web of Science. Details of the full electronic search strategy are available in the Supplementary Material, S1, available at https://doi.org/10.1016/j.esmogo.2025.100167. The search was limited to English studies conducted in humans. After eliminating duplicate citations, two independent researchers (AM and SV) evaluated the remaining articles based on title and abstract. The full texts of the remaining articles were reviewed by the same authors, with any disagreements resolved through discussion. If consensus could not be reached, a third reviewer (AK) was consulted. Studies were included if patients with primary head and neck squamous cancer (HNC), primary esophageal squamous cancer (EC) or primary lung squamous cancer (LC) were screened for SPTs in any of these sites. Studies were excluded if no full text was available, when screening method was unknown or if the study was conducted in an Asian population. Case reports and reviews were also excluded.

### Assessment of study quality

The Methodological Index for Non-Randomized Studies (MINORS) was used to assess the risk of bias and the methodological quality of the selected studies.^17^ The relevance of each study was evaluated using a checklist that considered (i) the impact factor of the publishing journal, which indicates peer-review quality, (ii) specific data on the SPT sublocation and (iii) the clarity of the text (Table 1). The overall quality score for each study was determined by adding the MINORS score to the relevance criteria score. These total scores were then categorized as low (≤10 points), medium (11-14 points), or high (≥15 points). Only studies that fell into the medium or high categories were included in the final analysis.

**Table 1:** Relevance criteria

Criteria	Score		
	0	1	2
Impact factor	<2	2-3.9	≥4
Sub-location	No	—	Yes
Text clarity	Low	Medium	High

### Data extraction and outcome parameters

**Figure 1:**
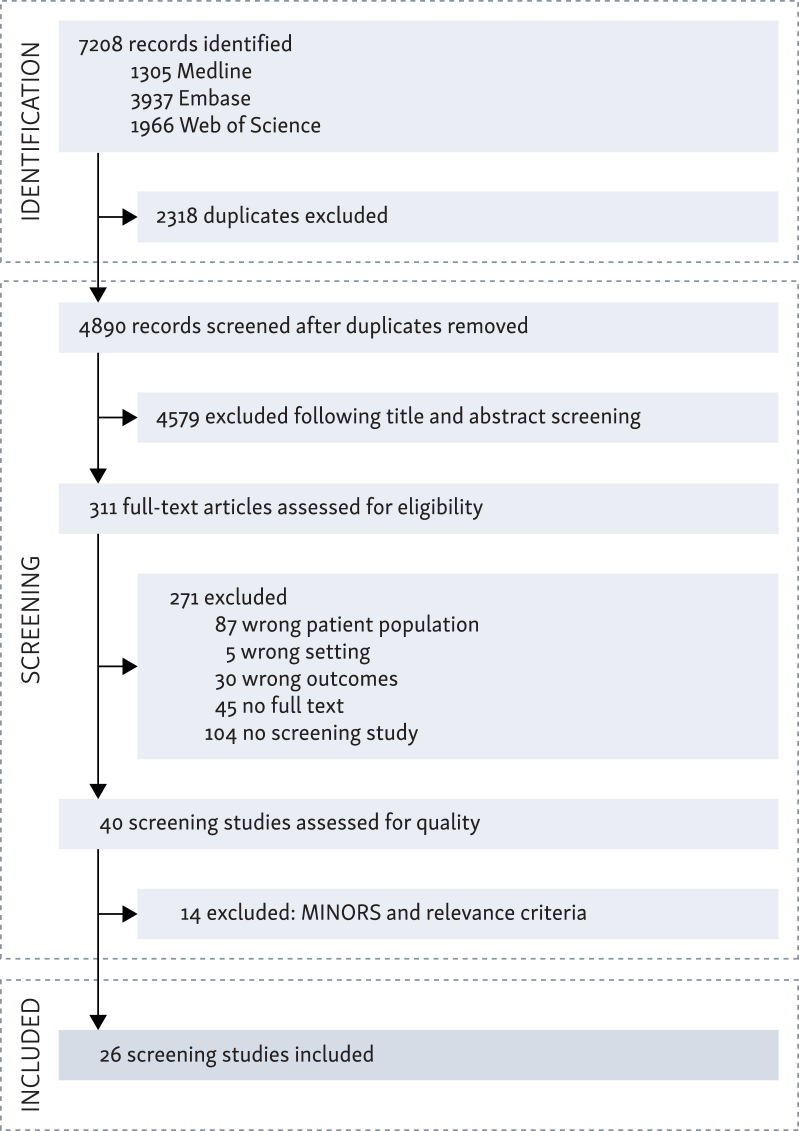
**Study selection process, Preferred Reporting Items for Systematic Reviews and Meta-Analyses flowchart**. MINORS, Methodological Index for Non-Randomized Studies.

Data from the included studies are summarized as a Preferred Reporting Items for Systematic Reviews and Meta-Analyses (PRISMA) checklist and flowchart^18^ (Figure 1). The primary outcome was the prevalence by screening of all diagnosed SPTs in the esophagus, head and neck or lungs. An SPT was defined as a lesion consistent with squamous cell cancer in the head and neck region, esophagus or lung according to the Warren and Gates criteria: (i) both primary and second primary must be malignant, (ii) both tumors must be anatomically separate and (iii) the possibility of the SPT being a metastasis from the index tumor must be excluded.^4^ Secondary outcomes were recorded when possible: method of screening (e.g. radiology, endoscopy, bronchoscopy), SPT prevalence per sublocation (HNC, EC and LC) and tumor stage, SPTs found synchronously (≤6 months after diagnosis of primary tumor) or metachronously (>6 months after diagnosis of primary tumor) and stage of SPT. Additionally, first author, country of study population, year of publication, study design, method of screening and population size were recorded.

### Statistical analysis

Counts and percentages were used to present the data. The prevalence of SPTs was determined for each study by dividing the total number of identified SPTs by the population that underwent screening in that study. For studies in which the standard error (SE) was not provided, we calculated it using the formula SE = √p(1−p)/*n*, where p represents the prevalence and *n* is the total number of patients with a primary tumor who were screened for HNC, EC and/or LC SPTs. Meta-analysis was conducted using R software (version 2023.12.0), using a random effects model to estimate the pooled prevalence. The *I*^2^ statistic was applied to assess heterogeneity across studies, and subgroup analyses were carried out based on specific tumor sublocation, stage and timing of screening (synchronously or metachronously).

## Results

### Study selection, quality assessment and characteristics

The process of our search query for eligible studies on SPT screening after HNC, EC or LC is presented in Figure 1. The search identified a total of 6812 studies. After removal of duplicates and title/abstract screening, 311 studies were reviewed for full-text screening by two researchers (AM and SV). Only non-Asian studies were included, originating from the USA, France, Germany, the Netherlands, Brazil, Poland or the UK. Discrepancies were discussed mutually, and no disagreements were reached. No studies focusing on screening for SPTs following a primary esophageal tumor were identified. One study addressed screening for SPTs after a primary lung tumor, while 32 studies focused exclusively on SPT screening following a head and neck tumor. Additionally, two studies investigated screening for SPTs following either a primary lung tumor or head and neck tumor. Due to the lack of data on screening after primary esophageal tumors and limited data on screening after primary lung tumors, the focus of this review was shifted to screening for SPTs following only primary head and neck tumors. Descriptive information on available lung tumor data is presented in the Supplementary Material S2, Supplementary Tables S1 and S2, available at https://doi.org/10.1016/j.esmogo.2025.100167. In total, 26 studies met the inclusion criteria for this systematic review.^19^, ^20^, ^21^, ^22^, ^23^, ^24^, ^25^, ^26^, ^27^, ^28^, ^29^, ^30^, ^31^, ^32^, ^33^, ^34^, ^35^, ^36^, ^37^, ^38^, ^39^, ^40^, ^41^, ^42^, ^43^, ^44^ A comprehensive overview of these studies is presented in Table 2. An overview of the excluded articles based on the quality scores is presented in the Supplementary Material S2, Supplementary Table S3, available at https://doi.org/10.1016/j.esmogo.2025.100167.

**Table 2:** Study characteristics and quality scores of the 26 included non-Asian screening studies for patients with an index head and neck tumor

Authors	Year	Design	*N*	Screening sites	Method	Quality score	Rel	Total	Quality
						MINORS			
Parker and Hill^19^	1988	Pro	208	EC, HNC, LC	Panendoscopy^a^	12	4	16	High
Shaha et al.^20^	1988	Pro	200	LC	Chest X-ray	11	2	13	Medium
Tan et al.^36^	1999	Pro	20	LC	CT + chest X-ray	10	3	13	Medium
Engelen et al.^21^	1992	Retro	556	LC	Chest X-ray	8	3	11	Medium
Petit et al.^22^	2001	Retro	1560	EC	Gastroscopy	9	3	12	Medium
Scherubl et al.^23^	2002	Pro	148	EC	Gastroscopy	11	6	17	High
Merkx et al.^24^	2002	Retro	339	LC	Chest X-ray	8	3	11	Medium
O’Meara et al.^25^	2003	Retro	78	LC	Chest X-ray	9	4	13	Medium
Hashimoto et al.^26^	2005	Pro	326	EC	Gastroscopy	11	4	15	High
Dubuc et al.^27^	2006	Pro	392	EC	Gastroscopy	10	4	14	Medium
Moschler et al.^28^	2006	Pro	87	EC	Gastroscopy	12	2	14	Medium
Jackel et al.^29^	2007	Retro	260	LC	Chest X-ray	9	3	12	Medium
Boller et al.^30^	2009	Pro	40	EC	Gastroscopy	11	3	14	Medium
Beech et al.^31^	2010	Retro	239	LC	CT	9	4	13	Medium
Farwell et al.^32^	2010	Pro	100	EC, HNC	TNE	10	2	12	Medium
Jaspers et al.^40^	2011	Retro	142	LC	CT	9	5	14	Medium
Wolff et al.^33^	2013	Pro	118	EC, HNC, LC	Gastroscopy, fiberscopy, CT	12	2	14	Medium
Nugent et al.^34^	2016	Retro	413	LC	CT	9	2	11	Medium
Halpenny et al.^35^	2016	Retro	26	LC	CT	10	4	14	Medium
Piersiala et al.^37^	2020	Retro	151	LC	CT	8	5	13	Medium
O’Dwyer et al.^38^	2021	Retro	105	LC	CT	9	4	13	Medium
Cramer et al.^39^	2021	Retro	171	LC	CT + chest X-ray	9	4	13	Medium
Van de Ven et al.^41^	2021	Pro	92	EC	Gastroscopy	12	5	17	High
Nobre Moura et al.^42^	2022	Retro	1888	EC	Gastroscopy	8	4	12	Medium
Van Tilburg et al.^43^	2023	Pro	202	EC	Gastroscopy	12	6	18	High
Chaber-Ciopinska et al.^44^	2023	Pro	294	EC	Gastroscopy	22	3	25	High

EC, esophageal cancer; HNC, head and neck cancer; LC, lung cancer; MINORS, Methodological Index for Non-Randomized Studies; *N*, number of patients with head and neck cancer included; Pro, prospective; Rel, relevance criteria; Retro, retrospective; TNE, transnasal endoscopy.

a Laryngoscopy, bronchoscopy, esophagoscopy.

### Prevalence of SPTs (synchronous and metachronous)

**Figure 2:**
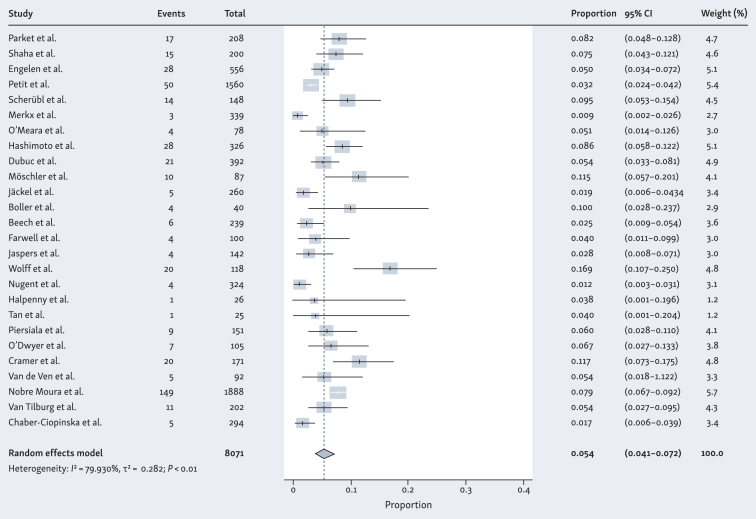
**Forest plot of prevalence of second primary tumors (esophagus, head and neck, lung) in patients with primary head and neck cancer.** CI, confidence interval; I^2^, inconsistency index; τ^2^, between-study variance.

Figure 2 illustrates the prevalence of esophageal, head and neck and lung SPTs combined across the studies included in patients with a primary head and neck tumor, ranging from 1.2% to 16.9%. A total of 445 SPTs were diagnosed by screening of 8071 patients. Due to an *I*^2^ value of 79.9%, a meta-analysis using a random-effects model was conducted to determine the pooled prevalence. The pooled prevalence for all SPTs in patients with a primary head and neck tumor across the 26 included studies was 5.4% (95% CI 4.1% to 7.2%) (Figure 2).

When analyzing the sublocations of SPTs individually, the prevalence of esophageal SPTs varied from 1.7% to 11.5%, of head and neck SPTs from 1.0% to 5.9% and of lung SPTs from 0.9% to 11.7% (Supplementary Material S3, Supplementary Figures S1, S2 and S3, available at https://doi.org/10.1016/j.esmogo.2025.100167). The pooled prevalence of esophageal, head and neck and lung SPTs separately was 5.3% (95% CI 3.7% to 7.7%), 4.6% (95% CI 1.0% to 18.1%) and 4.0% (95% CI 2.6% to 6.2%), respectively.

### Method of screening

All 307 SPTs in the esophagus and all 18 SPTs in the head and neck region were detected by endoscopy. Screening for SPTs in the lungs resulted in the detection of 120 tumors. Of these, 1.5% (*n* = 3) were identified by bronchoscopy, 52.9% (*n* = 63) by chest X-ray and 44.5% (*n* = 53) by chest computed tomography (CT). In one study, both X-ray and CT imaging were utilized, both confirming the presence of an SPT of bronchial origin.

### Prevalence per index tumor sublocation and stage

Sublocation of the index head and neck tumor was reported in 20 of 26 studies.^19^^,^^21^, ^22^, ^23^, ^24^^,^^26^^,^^27^^,^^29^^,^^31^, ^32^, ^33^, ^34^^,^^36^^,^^37^^,^^39^, ^40^, ^41^, ^42^, ^43^, ^44^ Of these 20 studies, 8 provided adequate reporting on the prevalence of SPTs per index head and neck sublocation.^21^^,^^24^^,^^29^^,^^36^^,^^40^^,^^41^^,^^43^^,^^44^ In these studies, a total of 65 SPTs were found through screening, of which 24 were esophageal and 41 lung SPTs. Overall, most tumors presented in combination with index hypopharynx tumors (*n* = 39, 60.0%), followed by oropharynx (*n* = 11, 16.9%), oral cavity (*n* = 9, 13.8%) and larynx (*n* = 6, 9.2%) tumors. Consistent with this finding, hypopharyngeal cancer was the most prevalent index tumor site for both esophageal and lung SPTs individually (42% and 71%, respectively). The least prevalent index tumor site varied between the two SPT sublocations: oral cavity cancer for esophageal SPTs (8%) and oropharynx cancer for lung SPTs (5%).

Stage of primary HN tumor has been described using varying classification systems over the years and only four studies presented the number of SPTs per primary tumor stage. Due to these differences and the limited data available, no adequate analysis was possible. Descriptive information of the four studies is presented in the Supplementary Material S2, Supplementary Table S4, available at https://doi.org/10.1016/j.esmogo.2025.100167.

### Stage of SPT

Stage of SPT was described using varying classification systems over the years conform the before mentioned paragraph on stage for index tumors. Survival data were lacking or not adequately reported in most studies. In two studies on screening for esophageal SPTs, tumor staging could be categorized according to the Vienna classification.^45^ Most (75%) were categorized as stage 4, comprising eight cases of high-grade dysplasia (HGD) and four cases of intramucosal carcinoma.^41^^,^^43^ Staging of lung SPTs was reported in three studies; however, the specific type and edition of the staging system used were not specified.^29^^,^^39^^,^^40^ Additionally, no data were available for tumor staging of head and neck SPTs. Consequently, a comprehensive analysis for lung or head and neck SPTs could not be conducted.

### Time to diagnosis

Ten studies analyzed both synchronous and metachronous screening,^20^^,^^21^^,^^23^^,^^25^^,^^32^^,^^33^^,^^35^^,^^38^^,^^42^^,^^43^ however only four studies provided detailed information on the screening rates for synchronous or metachronous SPTs^20^^,^^25^^,^^42^^,^^43^ (Table 3). The median time to diagnosis was reported in only one study screening for esophageal SPTs alone and was 0 months with a range from 0 to 32 months for a total of 11 SPTs.^42^^,^^43^

**Table 3:** Percentages of synchronous and metachronous SPTs for patients with an index head and neck tumor

Authors	Total SPTs	Synchronous SPTs (%)	Metachronous SPTs (%)	Median time to diagnosis, months (range)
Shaha et al.^20^	15	9 (60)	6 (40)	Not reported
O’Meara et al.^25^	4	0 (0)	4 (100)	Not reported
Nobre Moura et al.^42^	149	66 (44.3)	83 (55.7)	Not reported
Van Tilburg et al.^43^	11	6 (54.5)	5 (45.5)	0 (0-32)
Total	179	81 (45.3)	98 (54.7)	

SPT, second primary tumor.

Seven studies carried out exclusively synchronous screening^19^^,^^28^^,^^29^^,^^34^^,^^36^^,^^40^^,^^41^ and five studies carried out exclusively metachronous screening.^22^^,^^24^^,^^30^^,^^37^^,^^44^ The median time to diagnosis for synchronous screening was only reported in the study by van de Ven et al.^41^ on screening for esophageal SPTs and was 9 days with a range of 6–20 days. For metachronous screening, the time to diagnosis ranged from 7 months to 12 years with a median of 43 months.^22^^,^^24^^,^^30^^,^^37^^,^^44^ Timing of screening was unknown for the remaining four studies.

## Discussion

HNC survivors have a higher mortality risk compared with the general healthy population, a risk that is partially attributable to the development of SPTs due to the ‘theory of field cancerization’.^46^ Based on the 26 included studies about SPT screening in patients with HNC, the pooled prevalence for SPTs in head and neck, esophagus or lungs combined was 5.4% (95% CI 4.1% to 7.2%). The separate pooled prevalence for esophageal SPTs was 5.1%, for head and neck SPTs 4.6% and for lung SPTs it was 4.0%. The majority of SPTs (60.0%) developed in combination with primary hypopharyngeal cancer, with similar proportions of synchronous (45.3%) and metachronous (54.7%) SPTs observed across all SPT groups.

### Asian versus non-Asian populations

A significant advantage of this systematic review, which only includes non-Asian studies, is the possibility to compare its findings with those of previous reviews conducted in mainly Asian populations. A systematic review, published in 2019, focused specifically on screening for esophageal SPTs using Lugol chromoendoscopy in patients with HNC.^47^ This review predominantly included studies from Asia (12 of 15 studies), involving a total of 3386 HNC patients. The pooled prevalence for esophageal SPTs for the Asian studies was 13.6% which is more than double the prevalence observed in the non-Asian studies included in this review. These regional disparities in SPT rates may be attributed to the higher overall incidence of SCC in Asian populations, likely due to greater exposure to risk factors such as smoking and alcohol consumption.^48^ It may also be explained by the fact that screening for SPTs is more comprehensively addressed and emphasized in Asian guidelines compared with non-Asian guidelines.^13^^,^^14^ A potential conclusion from this observation is that screening for SPTs may be particularly warranted in Asian populations compared with regions outside of Asia. The studies included in this review, as well as those in the review by Bugter et al.,^47^ however, do not specify the composition of their populations. As a result, Asian and non-Asian patients may have been included in both analyses. Consequently, a definitive conclusion regarding differences between ethnic groups cannot be drawn with certainty.

Notably, the risk for EC following primary HNC is significantly higher than the risk for esophageal adenocarcinoma in patients with Barrett’s esophagus (BE).^49^^,^^50^ BE, characterized by metaplasia of the squamous cell mucosa into columnar epithelium, can progress to dysplasia and eventually adenocarcinoma.^50^ Surveillance for BE is widely accepted and recommended in multiple guidelines worldwide.^51^^,^^52^ Given the pooled prevalence of SPTs in >1 in 20 HNC survivors, as identified in our systematic review, the universal importance of implementing screening for SPTs is underscored, extending beyond geographic regions.

### Screening versus non-screening

Through analyzing retrospective non-screening studies conducted outside of Asia, some valuable insights were provided. These studies reported the prevalence of SPTs in esophagus, lungs and head and neck in patients with primary HNC and found this to be up to 17%,^15^^,^^16^ which is similar to the highest prevalence reported in the included studies in this review (0.9%-16.9%). When evaluating these non-screening studies for the different sublocations separately, it is noteworthy that the prevalence for esophageal SPTs is considerably lower compared with our included studies: 0.9% and 1.9% versus 5.3%.^15^^,^^16^ Analogously, this phenomenon has also been reported in the systematic review by Bugter et al.^47^ For lung and head and neck SPTs these numbers do not significantly differ between screening and non-screening studies.^15^^,^^16^ Overall, this may suggest that the implementation of active screening in HNC patients could lead to an increased detection rate of esophageal SPTs.

### Sublocation of index HNC

Several researchers aimed to identify the sublocation of index tumors in head and neck malignancies that might have a higher incidence of SPTs. Milano et al.^53^ and Adjei Boakye et al.^54^ analyzed 61 883 and 109 512 patients with HNC, respectively, from the Surveillance, Epidemiology, and End Results (SEER) database. While Milano et al.^53^ focused only on development of lung SPTs after HNC, Adjei Boakye et al.^54^ reported standardized incidence ratios of multiple different sites of SPTs, including head and neck, esophagus and lung. In addition, two Taiwanese retrospective studies studied the risk per sublocation of index HNC for the development of esophageal or lung SPTs.^55^^,^^56^ All four studies concluded that tumors located in the hypopharynx were the most commonly associated index site of HNC in the development of SPTs, which corroborates our findings.

### Stage of SPT

The studies in our review that reported on stage of esophageal SPTs showed that half of all SPTs detected by screening were HGD. Among the remaining lesions classified as carcinoma, 62.5% were superficial (T1). Although data were available from only two studies, it is important that most lesions were found at an early stage. Early-stage carcinomas have a lower risk of metastasis to lymph nodes or distant organs compared with those at advanced stages.^57^^,^^58^ This opens the possibility for minimally invasive endoscopic treatment, which can improve patient outcomes by avoiding more invasive procedures such as esophagectomy, which is associated with higher morbidity and mortality.^59^^,^^60^ Moreover, survival rates are substantially higher for early-stage cancers,^61^ further underlining the importance of early diagnosis and screening. We would advocate that future screening studies adequately report the stage of SPTs, as this will provide additional information on the potential impact of screening on staging.

### Timing of screening

Analyzing differences in follow-up time proved challenging in this review, due to insufficient data provided by most studies. In one study that screened both synchronously and metachronously for esophageal SPTs exclusively, the median time to diagnosis was adequately reported and was 0 months with a range of 0-32 months.^42^^,^^43^ Differences in timing of screening might have influenced the reported rates of SPTs as the cumulative incidence of SPTs increases each year.^5^^,^^62^ It is important that screening will be implemented in the group of patients that may benefit from it. The study conducted by van Tilburg et al.^43^ reported that 25% of all eligible patients had to be excluded due to being diagnosed with incurable stages of primary HNC within the first year of diagnosis. Consequently, timing of screening should be contingent upon the curative potential of the primary HNC. Yet, the research group reported that willingness to undergo screening was strongly dependent on timing of screening. The participation rate for endoscopic screening decreased from 90% during HNC work-up to ∼50% ≥2 years after follow-up.^43^ Determining an optimal window for screening, which includes patients with the highest chances of survival and a high willingness to undergo screening, is challenging, but may be best estimated between 1 or 2 years following curative treatment of HNC. It remains, however, an area of uncertainty that needs further exploration.

### Type of screening

Over the past years, screening and surveillance methods for different types of cancer have evolved. Flexible nasal fiberoptic endoscopy has been the most commonly used method of screening for HNC, while bronchoscopy, a chest radiograph or CT are more common for LC as demonstrated by the studies included in this review.^63^^,^^64^ For EC, gastroscopy has been the cornerstone of diagnosis for many years with the use of Lugol dye or image-enhanced endoscopy techniques, such as narrowband imaging (NBI), as most recent developments.^65^^,^^66^ All studies on esophageal SPTs included in this review used gastroscopy as screening method. (Diagnostic) gastroscopies are universally carried out with a low risk of complications, of which the most common are bleeding and perforation in 0-0.03% of procedures.^67^^,^^68^ Since studies have demonstrated that gastroscopy significantly increases the probability of detecting cancer and facilitates biopsy collection for histological confirmation of the diagnosis, other screening modalities, such as chest radiographs, have largely been abandoned.^69^^,^^70^ A notable observation from our included studies is that the introduction of the endoscopic modality NBI in 2005 has led to an increase in the mean prevalence of esophageal SPTs detected through screening, from 4.2% to 6.1%. Furthermore, a 2020 review indicated that the use of Lugol’s solution during screening for EC is increasingly being abandoned, as NBI appears to offer superior specificity and characterization of lesions.^71^ This indicates that this innovative technique contributes to a higher detection of SPTs in the esophagus.

For patients with HNC, [^18^F]-fluoro-2-deoxy-d-glucose positron emission tomography/CT (^18^F-FDG PET/CT) and even PET/MRI have a well-defined role in pretreatment staging and treatment response assessment.^72^ This is due to important technological innovations providing quick and high-resolution imaging.^72^ The question arises whether these imaging modalities could be the future replacement for the before mentioned screening methods for SPTs. The study by Haerle et al.^73^ comparing ^18^F-FDG PET/CT with panendoscopy (rigid tracheobronchoscopy, rigid esophagoscopy, direct laryngoscopy and hypopharyngoscopy) showed that ^18^F-FDG PET/CT might be superior to panendoscopy due to a higher sensitivity (100% versus 74%) and negative predictive value (98% versus 100%). They concluded that with a negative ^18^F-FDG PET/CT, the extent of endoscopy can be reduced to the area of the index HNC.^73^ Due to the substantial costs associated with an ^18^F-FDG PET/CT, however, along with the additional costs and patient burden resulting from the high number of false-positive findings, routine screening with ^18^F-FDG PET/CT or PET/MRI is currently still not recommended.^73^

### Current practice

Today, both American and European guidelines give no specific recommendations on standard screening for SPTs in the triad mentioned in our review. The American Cancer Society Head and Neck Cancer Survivorship Care Guideline suggests screening for lung cancer according to the American Society of Clinical Oncology (ASCO) or National Comprehensive Cancer Network (NCCN) recommendations for annual lung cancer screening with low-dose CT for high-risk patients based on smoking history.^74^ Furthermore, the guideline recommends screening for head and neck or esophageal SPT in patients with increased risk without describing these specific risks. The European Society for Medical Oncology (ESMO) states that prevention and screening for cancers other than HNC that share the same risk factors should be carried out according to their respective guidelines.^75^

### Conclusions and recommendations

As treatment strategies for HNC may further improve over time, the number of HNC survivors who are at risk for development of an SPT is expected to rise accordingly. Most SPTs in HNC survivors are detected in advanced stages resulting in a poor prognosis with a median survival of 12 months after SPT diagnosis.^76^ Therefore, early detection of SPTs through screening might be recommended for certain patient populations. Screening for lung or head and neck SPTs is not unequivocally recommended, as this review did not clarify the added value of screening compared with results from non-screening studies. In contrast, endoscopic screening for esophageal SPTs in non-Asian countries should be considered, especially in patients with a primary hypopharynx carcinoma, as results demonstrated an increased tumor detection rate at an early stage when compared with non-screening studies. Additionally, prospective, large-scale studies outside of Asia are needed to further investigate the potential benefits and optimal timing of screening for SPTs, particularly in the esophagus, as this may offer the greatest added value within the aerodigestive tract. Moreover, these studies should aim to identify risk factors associated with the development of SPTs, facilitating the creation of individualized risk profiles. Such targeted screening in specific populations is likely to be more cost-effective.
