# Supplementary material for: Screening for second primary tumors in the aerodigestive tract in non-Asian populations with head and neck cancer – systematic review and meta-analysis
Source: ESMO Gastrointest Oncol. 2025 Apr 3;8:100167. doi: 10.1016/j.esmogo.2025.100167 (PMC12836664; doi:10.1016/j.esmogo.2025.100167)

**Supplementary material**

**S1. Search strategy**

*Medline*

(exp Esophageal Neoplasms / OR ((esophag* OR oesophag* OR upper-aerodigest* OR upper-digest*) ADJ6 (tumo* OR malignan* OR carcin* OR adenocarcin* OR neoplas* OR cancer*)).ab,ti,kw.) AND (exp Head and Neck Neoplasms / OR Laryngeal Neoplasms / OR exp Lung Neoplasms / OR ((lip OR mouth OR oral OR nose OR nasal OR tongue OR tonsil OR nasopharyn* OR oropharyn* OR hypopharyn* OR pharyn* OR laryn* OR head OR neck OR pulmonary* OR lung*) ADJ6 (tumo* OR malignan* OR carcin* OR adenocarcin* OR neoplas* OR cancer*)).ab,ti,kw.) AND (Mass Screening / OR Early Detection of Cancer / OR (screening OR early-detect* OR early-diagnos*).ab,ti,kw.) AND english.la. NOT (exp animals/ NOT humans/)

(exp Head and Neck Neoplasms / OR Laryngeal Neoplasms / OR ((lip OR mouth OR oral OR nose OR nasal OR tongue OR tonsil OR nasopharyn* OR oropharyn* OR hypopharyn* OR pharyn* OR laryn* OR head OR neck) ADJ6 (tumo* OR malignan* OR carcin* OR adenocarcin* OR neoplas* OR cancer*)).ab,ti,kw.) AND (exp Lung Neoplasms / OR ((pulmonary* OR lung*) ADJ6 (tumo* OR malignan* OR carcin* OR adenocarcin* OR neoplas* OR cancer*)).ab,ti,kw.) AND (Mass Screening / OR Early Detection of Cancer / OR (screening OR early-detect* OR early-diagnos*).ab,ti,kw.) AND english.la. NOT (exp animals/ NOT humans/)

*Embase*

('esophagus tumor'/exp OR ((esophag* OR oesophag* OR upper-aerodigest* OR upper-digest*) NEAR/6 (tumo* OR malignan* OR carcin* OR adenocarcin* OR neoplas* OR cancer*)):ab,ti,kw) AND ('head and neck tumor'/exp OR 'larynx tumor'/exp OR 'lung tumor'/exp OR ((lip OR mouth OR oral OR nose OR

nasal OR tongue OR tonsil OR nasopharyn* OR oropharyn* OR hypopharyn* OR pharyn* OR laryn* OR head OR neck OR pulmonary* OR lung*) NEAR/6 (tumo* OR malignan* OR carcin* OR adenocarcin* OR neoplas* OR cancer*)):ab,ti,kw) AND (screening/de OR 'cancer screening'/de OR 'early cancer diagnosis'/de OR (screening OR early-detect* OR early-diagnos*):ab,ti,kw) AND [english]/lim NOT ([animals]/lim NOT [humans]/lim) NOT ([conference abstract]/lim)

('head and neck tumor'/exp OR 'larynx tumor'/exp OR ((lip OR mouth OR oral OR nose OR nasal OR tongue OR tonsil OR nasopharyn* OR oropharyn* OR hypopharyn* OR pharyn* OR laryn* OR head OR neck) NEAR/6 (tumo* OR malignan* OR carcin* OR adenocarcin* OR neoplas* OR cancer*)):ab,ti,kw) AND ('lung tumor'/exp OR ((pulmonary* OR lung*) NEAR/6 (tumo* OR malignan* OR carcin* OR adenocarcin* OR neoplas* OR cancer*)):ab,ti,kw) AND (screening/de OR 'cancer screening'/de OR 'early cancer diagnosis'/de OR (screening OR early-detect* OR early-diagnos*):ab,ti,kw) AND [english]/lim NOT ([animals]/lim NOT [humans]/lim) NOT ([conference abstract]/lim)

*Web of science*

TS=((((esophag* OR oesophag* OR upper-aerodigest* OR upper-digest*) NEAR/5 (tumo* OR malignan* OR carcin* OR adenocarcin* OR neoplas* OR cancer*))) AND (((lip OR mouth OR oral OR nose OR nasal OR tongue OR tonsil OR nasopharyn* OR oropharyn* OR hypopharyn* OR pharyn* OR laryn* OR head OR neck OR pulmonary* OR lung*) NEAR/5 (tumo* OR malignan* OR carcin* OR adenocarcin* OR neoplas* OR cancer*))) AND ((screening OR early-detect* OR early-diagnos*))) NOT DT=(Meeting Abstract OR Meeting Summary) AND LA=(English)

TS=((((lip OR mouth OR oral OR nose OR nasal OR tongue OR tonsil OR nasopharyn* OR oropharyn* OR hypopharyn* OR pharyn* OR laryn* OR head OR neck) NEAR/5 (tumo* OR malignan* OR carcin* OR adenocarcin* OR neoplas* OR cancer*))) AND (((pulmonary* OR lung*) NEAR/5 (tumo* OR malignan* OR carcin* OR adenocarcin* OR neoplas* OR cancer*))) AND ((screening OR early-detect* OR early-diagnos*))) NOT DT=(Meeting Abstract OR Meeting Summary) AND LA=(English)

**S2. Supplementary tables**

Table 1. Study characteristics and quality scores of all 3 non-Asian screening studies for patients with an index lung tumor

|  |  |  |  |  |  | *Quality Score* |  |  |  |
| --- | --- | --- | --- | --- | --- | --- | --- | --- | --- |
| **Authors** | **Year** | **Design** | ***N*** | **Screening sites** | **Method** | **MINORS** | **Rel** | **Total** | **Quality** |
| Halpenny et al. | 2016 | Retro | 16 | LC | CT | 10 | 4 | 14 | Medium |
| O’Dwyer et al. | 2021 | Retro | 87 | LC | CT | 9 | 4 | 13 | Medium |
| **Exclusion** |  |  |  |  |  |  |  |  |  |
| Pasic et al. | 2003 | Retro | 18 | LC | Bronchoscopy | 7 | 3 | 10 | Low |

*N*: number of patients with lung cancer included; MINORS: Methodological Index for Non-Randomized Studies; Rel: relevance criteria; Pro: prospective; Retro: retrospective; LC: lung cancer.

Table 2. Number of SPTs per screening study for patients with an index lung tumor

| **Authors** | ***N*** | **SPTs** | **Rate** |
| --- | --- | --- | --- |
| Halpenny et al. | 16 | 0 | 0% |
| O’Dwyer et al. | 87 | 7 | 8% |
| Total | 103 | 7 | 7% |

*N*: number of patients with lung cancer included; SPT: second primary tumor

Table 3. Study characteristics and quality scores of the 13 excluded non-Asian screening studies for patients with an index head and neck tumor

|  |  |  |  |  |  | *Quality Score* |  |  |  |
| --- | --- | --- | --- | --- | --- | --- | --- | --- | --- |
| **Authors** | **Year** | **Design** | ***N*** | **Screening sites** | **Method** | **MINORS** | **Rel** | **Total** | **Quality** |
| Stalpers et al. | 1989 | Retro | 213 | HNC, LC | Chest X-ray | 8 | 2 | 10 | Low |
| Dhooge et al. | 1996 | Pro | 118 | EC, HNC, LC | Chest x-ray | 9 | 1 | 10 | Low |
| Mercader et al. | 1997 | Retro | 93 | EC, LC | CT | 9 | 1 | 10 | Low |
| Shah et al. | 2000 | Retro | 1086 | LC | Chest X-ray | 7 | 2 | 9 | Low |
| Tincani et al. | 2000 | Pro | 60 | EC | Gastroscopy | 7 | 2 | 9 | Low |
| Arunachalam et al. | 2002 | Pro | 44 | LC | CT + chest X-ray | 7 | 1 | 8 | Low |
| Loh et al. | 2005 | Retro | 102 | LC | CT + chest X-ray | 7 | 2 | 9 | Low |
| Leong et al. | 2008 | Retro | 102 | LC | Chest X-ray | 8 | 2 | 10 | Low |
| Ghosh et al. | 2009 | Retro | 1882 | LC | CT + chest X-ray | 7 | 1 | 8 | Low |
| Kesting et al. | 2009 | Retro | 570 | LC | Bronchoscopy | 6 | 4 | 10 | Low |
| Çetnkaya et al. | 2011 | Pro | 30 | LC | Bronchoscopy | 8 | 1 | 9 | Low |
| Kominek et al. | 2013 | Pro | 132 | EC | Gastroscopy | 7 | 2 | 9 | Low |
| Ozdemir et al. | 2020 | Retro | 183 | EC, HNC, LC | PET-CT, pharyngolaryngoscopy | 10 | 0 | 10 | Low |

*N*: number of patients with head and neck cancer included; MINORS: Methodological Index for Non-Randomized Studies; Rel: relevance criteria; Pro: prospective; Retro: retrospective; EC: esophageal cancer; HNC: head and neck cancer; LC: lung cancer;

Table 4. Number of second primary tumors per stage of primary head and neck tumor

| **Authors** | **Stage 0** | **SPT, *N* (%)** | **Stage 1** | **SPT, *N* (%)** | **Stage 2** | **SPT, *N* (%)** | **Stage 3** | **SPT, N (%)** | **Stage 4** | **SPT, *N* (%)** | **Stage Unclear** | **SPT, *N* (%)** |
| --- | --- | --- | --- | --- | --- | --- | --- | --- | --- | --- | --- | --- |
| Jaspers et al.* | 0 | 0 (0%) | 42 | 0 (0%) | 34 | 1 (2.9%) | 19 | 2 (10.5%) | 47 | 1 (2.1%) | 0 | 0 (0%) |
| Tan et al.** | 0 | 0 (0%) | 0 | 0 (0%) | 0 | 0 (0%) | 4 | 0 (0%) | 21 | 5 (23.8%) | 0 | 0 (0%) |
| Van de Ven et al.*** | 0 | 0 (0%) | 15 | 0 (0%) | 29 | 4 (13.8%) | 24 | 1 (4.2%) | 16 | 2 (12.5%) | 7**** | 0 (0%) |
| Van Tilburg et al.*** | 0 | 0 (0%) | 46 | 1 (2.5%) | 70 | 8 (11.4%) | 46 | 0 (0%) | 36 | 3 (8.3%) | 18**** | 0 (0%) |

*= The Union for International Cancer Control 2002 classification, 6^th^ edition
**= The Union for International Cancer Control 2016 classification, 8^th^ edition
***= T-stage
****= carcinoma in situ
SPT: second primary tumor; *N*: number of SPTs

**S3. Supplementary figures**

Figure 1. Forest plot of prevalence of second primary tumors per sublocation (esophagus) in patients with head and neck cancer. CI: confidence interval; I^2^: inconsistency index; τ^2^: between-study variance

*
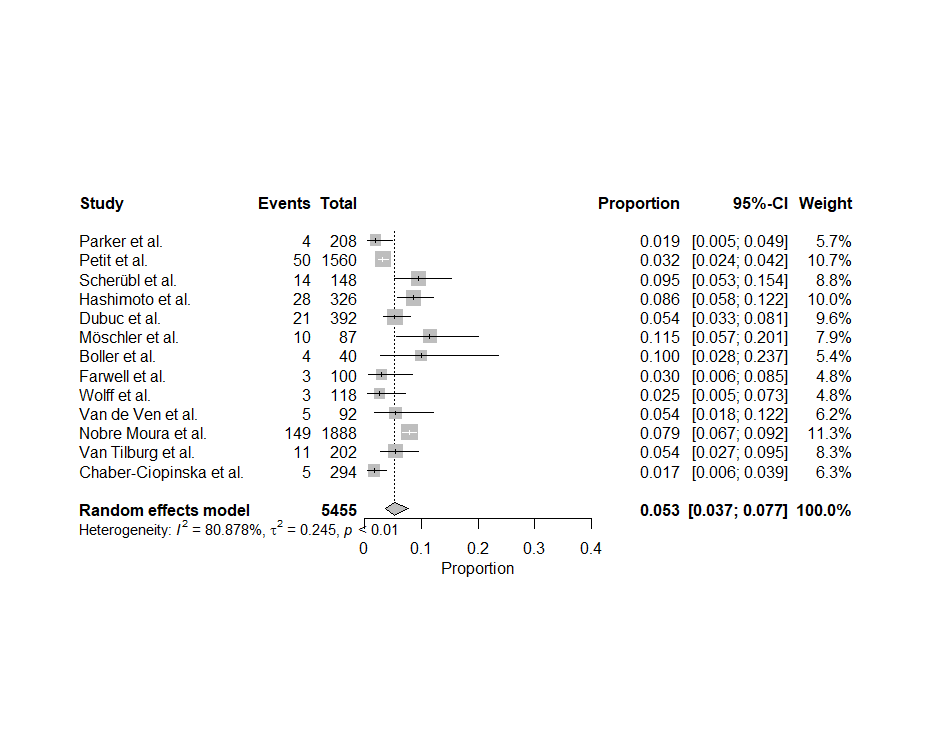
*

Figure 2. Forest plot of prevalence of second primary tumors per sublocation (head and neck) in patients with head and neck cancer. CI: confidence interval; I^2^: inconsistency index; τ^2^: between-study variance

*
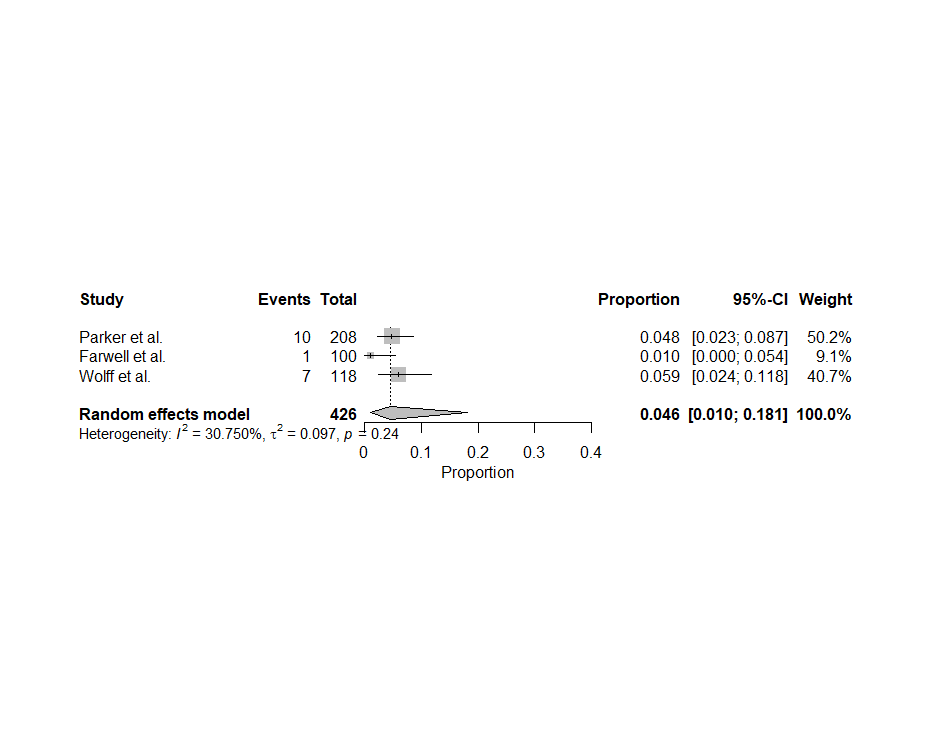
*

Figure 3. Forest plot of prevalence of second primary tumors per sublocation (lung) in patients with head and neck cancer. CI: confidence interval; I^2^: inconsistency index; τ^2^: between-study variance


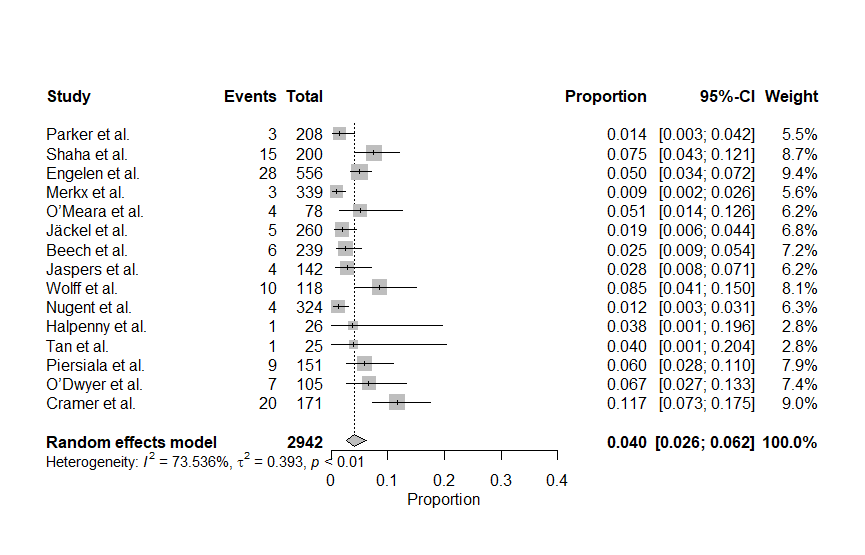

Supplement: Supplementary Material [file mmc1.docx]
